# Assessment of Vitamin D Status in Primary Hyperparathyroidism Patients: A Retrospective Study

**DOI:** 10.7759/cureus.64988

**Published:** 2024-07-20

**Authors:** Melinda Kolcsar, László Szabó, Orsolya Mária Dénes, Zsolt Gáll

**Affiliations:** 1 Department of Pharmacology and Clinical Pharmacy, George Emil Palade University of Medicine, Pharmacy, Science, and Technology of Târgu Mureș, Târgu Mureș, ROU; 2 Department of Endocrinology, George Emil Palade University of Medicine, Pharmacy, Science, and Technology of Târgu Mureș, Târgu Mureș, ROU

**Keywords:** 25-hydroxyvitamin d, parathyroid adenoma, vitamin d deficiency, hypercalcemic primary hyperparathyroidism, normocalcemic primary hyperparathyroidism

## Abstract

Background: Primary hyperparathyroidism (PHPT), a condition that manifests in various clinical forms, is a significant health concern. Normocalcemic primary hyperparathyroidism (NPHPT) is characterized by normal calcemia despite elevated parathyroid hormone (PTH) levels. Vitamin D deficiency can contribute to the clinical spectrum and complexity of NPHPT. Low vitamin D levels can elevate PTH, making it difficult to distinguish between NPHPT and secondary hyperparathyroidism. Additionally, it might mask hypercalcemia, leading to an underestimation of the disease severity. Our study aims to shed light on these complexities by investigating normocalcemic and hypercalcemic PHPT patient's clinical, hormonal, and biochemical patterns, including their vitamin D status.

Materials: In this retrospective study, we enrolled 60 PHPT patients with autonomous parathyroid function confirmed using a combination of ultrasonography, radionuclide scan, and parathyroid function index calculation. We evaluated the albumin-corrected calcemia, calciuria, PTH, 25(OH)D level, serum phosphate, bone mineral density, and major clinical symptoms (fracture, nephrolithiasis). A comparative analysis and a correlation study were performed between normo- and hypercalcemic and vitamin D-deficient and vitamin D-non-deficient groups.

Results: The median age was 62 years, 51.66% (31/60) being normocalcemic and 46.66% (29/60) presenting a deficient 25(OH)D level. In the group with 25(OH)D below 20 ng/mL, we observed a reduced level of albumin-corrected calcemia, without a significant increase of PTH compared to the adequate 25(OH)D level group. The frequency of the NPHPT and the risk of fracture were significantly higher in the deficient 25(OH)D group (20/60, 33.33% and 8/60, 13.33%) than in the adequate one (11/60, 18.33% and 1/60, 1.66%) with OR=4.7 (p<0.004) and OR=9.7 (p<0.027), respectively. We also found a positive correlation between PTH and adenoma size, the parathyroid function index and adenoma size, as well as PTH and phosphate levels. However, the correlation between 25(OH)D and phosphate levels was negative and moderate (rho=-0.504, p<0.001), adding a new layer of complexity to our understanding of these relationships.

Conclusion: Our study provided significant insight into the link between vitamin D status and normocalcemic PHPT. We found that vitamin D-deficient patients with normocalcemic PHPT have an increased fracture risk, which requires meticulous monitoring and possible supplementation with vitamin D. This should be done carefully to avoid exacerbating hypercalcemia or hypercalciuria. Further research is needed to refine these management strategies and deepen our understanding of the complex relationships between the analyzed parameters.

## Introduction

Primary hyperparathyroidism (PHPT) is characterized by the excessive and autonomous production of parathyroid hormone (PTH) by one or more parathyroid glands. These glands, located in the neck adjacent to the thyroid gland, play a crucial role in maintaining calcium and phosphate balance in the body. PTH acts directly via its receptors on two target organs, bones and kidneys, and indirectly via vitamin D receptors on the intestines to regulate calcium and phosphate levels. Vitamin D synthesis is enhanced by PTH, which stimulates the enzyme 1-alpha-hydroxylase in the kidneys, converting 25-hydroxyvitamin D (25(OH)D or calcidiol) into its active form, 1,25-dihydroxyvitamin D (1,25(OH)2D or calcitriol).  The interaction between calcitriol and PTH involves a complex feedback mechanism: the elevated blood calcium levels inhibit the secretion of PTH from the parathyroid glands through a negative feedback mechanism; calcitriol also has a direct suppressive effect on PTH gene expression in the parathyroid glands and directly inhibits the synthesis of 1-alpha-hydroxylase and stimulates the production of 24-hydroxylase, which converts calcitriol to inactive metabolites [1].

The etiology of PHPT primarily involves the following causes as a source of elevated PTH: parathyroid adenoma (85-90% of cases), parathyroid hyperplasia (10‑15% of cases, sporadically or as part of genetic syndromes), and parathyroid carcinoma (less than 1%). Classically, the overproduction of PTH leads to hypercalcemia through increased calcium release from bones, increased calcium reabsorption in the kidneys, and increased calcium absorption from the intestines. There have been several studies that suggest vitamin D deficiency (defined as a 25(OH)D level at least <20 ng/mL) is associated with a more severe presentation of PHPT. This is due to higher PTH levels, increased bone turnover markers, lower bone mineral density, and increased parathyroid mass [2-5]. PHPT can be classified into symptomatic and asymptomatic forms, with both forms further divided into hypercalcemic primary hyperparathyroidism (HPHPT) and normocalcemic primary hyperparathyroidism (NPHPT). In NPHPT patients, both normal ionized calcium and total albumin-corrected calcium levels are in the normal range, but generally with a low bone mass associated increase in PTH levels [6]. To evaluate the absence of hypercalcemia in NPHPT, secondary causes of hyperparathyroidism, such as vitamin D deficiency, chronic kidney disease, malabsorption, and medication use such as proton pump inhibitors, corticosteroids, lithium, antiepileptics, loop diuretics, bisphosphonates, and denosumab, must also be ruled out [7,8]. Therefore, interpreting insufficient or deficient 25(OH)D vitamin levels in the case of high PTH associated with normocalcemia is controversial.  

The aim of our study was to evaluate vitamin D status in patients with confirmed autonomous PTH secretion with or without hypercalcemia to assess the impact of different 25(OH)D levels and the high PTH on bone health and biochemical changes.

## Materials and methods

Study population**

This retrospective research included patients with elevated PTH levels associated with hypercalcemia or normocalcemia in which a technetium-99m sestamibi scan confirmed the parathyroid hyperfunction. From January 1, 2014, to January 31, 2019, clinical, biochemical, hormonal, and imagery data of the selected patients from the Department of Endocrinology of Mures County Clinic were recorded. All clinical symptoms and comorbidities, including nephrolithiasis, previous fractures, and dual-energy X-ray absorptiometry analysis (DXA) data, were documented by using medical history and a review of medical records. We defined asymptomatic subjects as those with biochemically present hyperparathyroidism but no overt signs of the disease (i.e., osteitis fibrosa cystica, nephrocalcinosis, reduced renal function, muscle weakness, peptic ulcer disease and pancreatitis, neurocognitive symptoms) according to Bilezikian et al. [9]. Subjects with any other condition that can affect bone and calcium metabolism, such as Paget disease, osteogenesis imperfecta, malabsorption diseases, thyrotoxicosis, chronic hepatic disease, hypercorticism, rheumatological and hematological diseases, and familial hypocalciuric hypercalcemia, were excluded. Administration of drugs affecting bone and calcium metabolism (proton pump inhibitors, diuretics, lithium, antiepileptics, bisphosphonates, denosumab, significant use of glucocorticoids within the past two years) were also considered criteria of exclusion. 

This study was conducted following the principles outlined in the Declaration of Helsinki. Ethical approval was obtained from the Ethics Committee of the George Emil Palade University of Medicine, Pharmacy, Science, and Technology of Târgu Mures (approval number: 747/18.02.2020).

Biochemical and hormonal analysis  

HPHPT was considered when serum PTH measured was above the reference range (normal: 15-65 pg/mL) associated with hypercalcemia (above 10.4 mg/dL considered the upper limit of the albumin-corrected calcemia), and NPHPT was considered if the PTH was elevated associated with normal calcium levels in condition of normal renal function, the estimated glomerular filtration rate (eGFR) being equal or higher than 90 mL/min/1.73 m² with values measured simultaneously and confirmed on at least two occasions. Albumin-adjusted calcium was used according to the following formula: albumin-corrected calcium (mg/mL)=total measured calcium+(0.8×(4-albumin)). eGFR was calculated using the recent chronic kidney disease epidemiology collaboration equation: eGFR (mL/min/1.73 m²)=175×(serum creatinine)-1.154×(age)-0.203×0.742 (if female).

High-performance liquid chromatography technique was used for all 25(OH)D level determinations in the same laboratory (S.C. Bioclinica SRL, Timișoara, Romania). A further precaution was taken to minimize seasonal variation by considering measurements obtained only between March and September excluding winter time decline of vitamin D. 25(OH)D levels were considered as defining the vitamin D status, sufficient when its value was between 30 and 100 ng/mL, insufficient when it was between 20 and 29 ng/mL, and deficient when it was below 20 ng/mL [10].

The recently introduced new parathyroid function index (PFi) as a new biomarker in differentiating PHPT from secondary forms [11] was used in our study. We applied the following formula: corrected calcium (mg/dL)×PTH (pg/mL)/phosphate (mg/dL).

Ultrasounds/sestamibi  

The enlarged parathyroid glands were identified and measured on neck ultrasound examination (Samsung HS60, 10-12 MHz probe, B-mode and Doppler, Samsung Healthcare, Danvers, Massachusetts, United States). A radiotracer (technetium-99m sestamibi) was used to identify hyperfunctioning parathyroid tissues and adenomas. The location of the adenomas and the craniocaudal and longitudinal diameters were noted, with the maximal diameter being used for statistical calculations.  

DXA

Validated DXA scans (Lunar scans, GE Healthcare, Madison, Wisconsin, United States) with T- and Z-scores reflecting bone mineral density (BMD, g/cm^2^) of the lumbar spine (L1‑L4), total hip, and left forearm were used. In order to account for the influence of gender and premenopausal status on bone health, the Z-score was used for premenopausal women and men under 50, while the T-score was used for postmenopausal women and men aged 50 or older. Osteoporosis was diagnosed when the T-score or Z-score fell below -2.5 standard deviations.

Statistical analysis  

All obtained data were analyzed using GraphPad Prism 10.2.3 (GraphPad Software Inc., San Diego, California, United States) and IBM SPSS Statistics for Windows, Version 27.0 (Released 2020; IBM Corp., Armonk, New York, United States). The Kolmogorov-Smirnov test was used to establish the sample distribution type. In the case of normally distributed variables, data are presented as the mean±SD, and in the case of non-normally distributed variables, the medians with interquartile range (IQR) are presented. The intergroup variables were compared with a t-test or the Mann-Whitney test as appropriate. Categorical variables are described as percentages, and intergroup comparisons were analyzed with the chi-squared test, Yates's chi-squared test, or Fisher's exact probability test. Correlations between variables were analyzed with the Spearman rank correlation coefficient. For correlation coefficient (rho) and odds ratio (OR), a 95% confidence interval (CI) was computed. In a binary logistic regression model predicting fractures, the relevance of age, PTH, albumin-corrected calcemia, and adenoma size were studied. A p-value <0.05 was considered statistically significant. 

## Results

Sixty patients fulfilled the criteria of PHPT confirmed by the laboratory analysis and the combined imagistic study. Their demographic, biochemical, clinical, and imagistic examination results are presented in Table 1.  

**Table 1:** Demographic, clinical, and paraclinical parameters of the study population IQR: interquartile range; SD: standard deviation; PTH: parathyroid hormone; PFi: parathyroid function index; eGFR: estimated glomerular filtration rate; 25(OH)D: 25-hydroxyvitamin D; DXA: dual-energy X-ray absorptiometry Normal values: albumin-corrected calcium: 8.5‑10.4 mg/dL, calciuria: 25‑300 mg/24 h in males, 20‑275 mg/24 h in females, phosphate: 2.5‑4.5 mg/dL, PTH:15‑65 ng/mL, 25(OH)D: 30‑100 ng/mL, eGFR above 90 mL/min/1.73 m^2^

Demographic characteristics of the patient population	
Total number	60
Female	58 (96.66%)
Male	2 (3.33%)
Age (year)	62 (IQR=5)
Biochemical parameters in patients with primary hyperparathyroidism	
Albumin-corrected calcium (mg/dL)	10 (±1.1 SD)
Calciuria (mg/24 h)	265 (IQR=138)
Phosphate (mg/dL)	2.4 (IQR=0.6)
PTH (pg/mL)	150 (IQR=127)
PFi	672 (IQR=571)
25(OH)D (ng/mL)	21 (IQR=18)
eGFR (mL/min/1.73 m²)	97 (IQR=11)
Clinical and paraclinical parameters in patients with primary hyperparathyroidism	
Parathyroid adenoma	51 (85%)
Parathyroid hyperplasia	9 (15%)
Nephrolithiasis	29 (48.33%)
Fractures confirmed	9 (15%)
DXA osteoporotic values	29 (48.33%)
Asymptomatic	11 (18.33%)

A statistical analysis was conducted based on the calcemia and vitamin D status of the included patients. According to their calcemia, patients were divided into NPHPT (n=31) and HPHPT (n=29) groups. The comparisons of demographic and biochemical parameters of NPHPT and HPHPT patients are presented in Table 2. 

**Table 2:** NPHPT and HPHPT patients' clinical, biochemical, and hormonal data NPHPT: normocalcemic primary hyperparathyroidism; HPHPT: hypercalcemic primary hyperparathyroidism; SD: standard deviation; IQR: interquartile range; PTH: parathyroid hormone; PFi: parathyroid function index; 25(OH)D: 25-hydroxyvitamin D; *: statistically significant; **: highly statistically significant Normal values: albumin-corrected calcium: 8.5‑10.4 mg/dL, calciuria: 25‑300 mg/24 h in males, 20‑275 mg/24 h in females, phosphate: 2.5‑4.5 mg/dL, PTH: 15‑65 ng/mL, 25(OH)D: 30‑100 ng/mL

	NPHPT (n=31, 51.66%)	HPHPT (n=29, 48.33%)	p	Test
Age (years); mean±SD, n=60	57.1±12	61.4±10	0.483	Independent samples t-test
Maximal diameter of adenoma (mm); mean±SD, n=51	9.4±2.2 (n=25)	9.9±3 (n=26)	0.513	Welch's corrected unpaired t-test
Albumin-corrected calcemia; mean±SD, n=60	9.56±0.49	11.37±0.6	<0.001**	Independent samples t-test
PTH (pg/mL); median (IQR), n=60	133 (IQR=55.1)	188.5 (IQR=188.9)	0.09	Mann-Whitney
Phosphate (mg/dL); mean±SD, n=60	2.4±0.3	2.4±0.3	0.634	Independent samples t-test
Calciuria (mg/24 h); mean±SD, n=60	259.2±125.5	281.4±111	0.956	Independent samples t-test
PFi; median (IQR), n=60	568 (IQR=279)	855 (IQR=888)	0.003**	Mann-Whitney
25(OH)D (ng/mL); median (IQR), n=60	13.75 (IQR=12.4)	22.9 (IQR=36.8)	0.045*	Mann-Whitney

**Figure 1:**
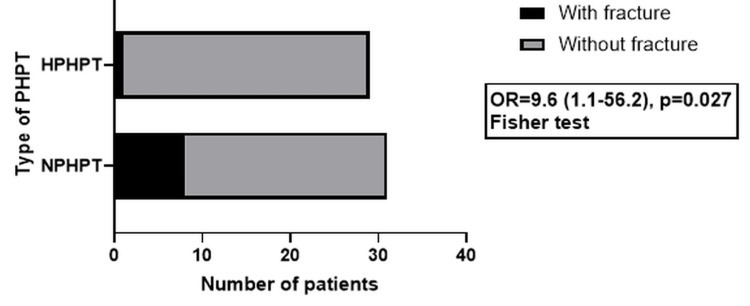
PHPT type influence on fracture risk PHPT: primary hyperparathyroidism; NPHPT: normocalcemic primary hyperparathyroidism; HPHPT: hypercalcemic primary hyperparathyroidism; OR: odds ratio

The association of clinical and paraclinical findings with calcemia was also investigated using the chi-squared or Fisher's exact probability test. No differences were found regarding the association of nephrolithiasis, osteoporotic DXA values, adenoma size, or location between NPHPT and HPHPT patients, but NPHPT patients had a higher fracture risk. The NPHPT-associated higher fracture risk is presented in Figure 1. 

According to their vitamin D status, patients were divided into vitamin D-deficient (<20 ng/ml) and adequate vitamin D (20‑100 ng/ml) groups. The demographic and biochemical data depending on the 25(OH)D levels are presented in Table 3. 

**Table 3:** Clinical, biochemical, and hormonal data of primary hyperparathyroidism patients depending on vitamin D status 25(OH)D: 25-hydroxyvitamin D; IQR: interquartile range; PTH: parathyroid hormone; PFi: parathyroid function index; *: statistically significant; **: highly statistically significant

	Deficient 25(OH)D level (<20 ng/mL) (n=28, 46.66%)	Adequate 25(OH)D level (20‑100 ng/mL) (n=32, 53.33%)	p	Test
Age (years), n=60	60 (IQR=8)	62 (IQR=11)	0.394	Mann-Whitney
Maximal diameter of adenoma (mm); median (IQR), n=51	9 (IQR=3), n=23	10 (IQR=3), n=28	0.131	Mann-Whitney
25(OH)D (ng/mL); median (IQR), n=60	12.9 (IQR=3.7)	30.4 (IQR=25.5)	<0.001**	Mann-Whitney
PTH (pg/mL); median (IQR), n=60	136.1 (IQR=201.5)	150 (IQR=139.5)	0.871	Mann-Whitney
Calcemia (mg/dL); mean±SD, n=60	10.2±0.9	10.7±1.05	0.017*	Independent samples t-test
Calciuria (mg/24 h); median (IQR), n=60	240.8 (IQR=143)	300.7 (IQR=147.4)	0.309	Mann-Whitney
Phosphate (mg/dL); median (IQR), n=60	2.5 (IQR=0.6)	2.2 (IQR=0.5)	0.003**	Mann-Whitney
PFi, n=60	595 (IQR=482)	704 (IQR=782)	0.23	Mann-Whitney

**Figure 2:**
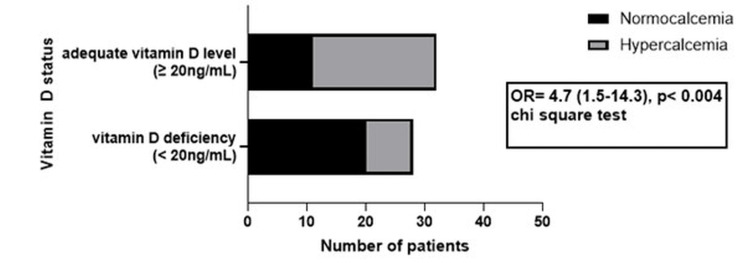
Vitamin D status influence on PHPT type PHPT: primary hyperparathyroidism; OR: odds ratio

The association of clinical features with vitamin D status was also evaluated. The risk of nephrolithiasis, osteoporotic DXA values, and adenoma size or location was not different among the patients with adequate or deficient 25(OH)D levels, but vitamin D deficiency significantly increased the risk for NPHPT and fracture. The increased risk for NPHPT in patients with vitamin D deficiency is presented in Figure 2. 

**Figure 3:**
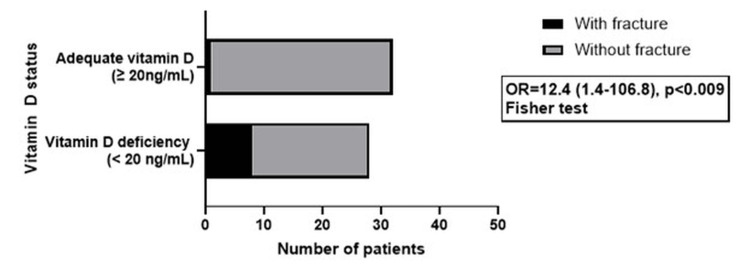
Vitamin D status influence on fracture risk OR: odds ratio

The increased risk for fracture in vitamin D-deficient PHPT patients is presented in Figure 3.

Our analysis revealed no correlation between the studied parameters, except for a significant positive linear correlation between PTH and phosphate levels, and the parathyroid adenoma size and PTH level, as well as adenoma size and PFi. Additionally, we found an inverse linear correlation between 25(OH)D level and serum phosphate, further confirming the precision of our findings. These results are presented in Table 4. 

**Table 4:** Correlation between age, PTH, PFi, albumin-corrected calcemia, calciuria, serum phosphate, vitamin D status, and parathyroid adenoma dimension rho: Spearman's correlation coefficient; 25(OH)D: 25-hydroxyvitamin D; PTH: parathyroid hormone; PFi: parathyroid function index; *: statistically significant; **: highly statistically significant ^1^Correlation was calculated for 51 patients with imagery confirmed adenoma

			Age (years)	PTH (pg/mL)	Albumin-corrected calcium (mg/dL)	Calciuria (mg/24 h)	Phosphate (mg/dL)	25(OH)D (ng/mL)	Adenoma max diameter (mm)^1^	PFi
Age (years)		rho	1.000	0.123	0.029	-0.001	0.076	0.134	0.120	0.119
		p		0.349	0.824	0.996	0.564	0.308	0.400	0.365
PTH (pg/mL)		rho	0.123	1.000	0.206	0.243	0.279 (0.019-0.503)	-0.099	0.728 (0.561-0.839)	0.921(0.869 -0.953)
		p	0.349		0.114	0.063	0.031*	0.453	<0.001**	<0.001
Albumin-corrected calcium (mg/dL)		rho	0.029	0.206	1.000	0.208	-0.010	0.161	0.053	0.416(0.173-0.61)
		p	0.824	0.114		0.114	0.940	0.220	0.712	<0.001
Calciuria (mg/24 h)		rho	-0.001	0.243	0.208	1.000	0.083	0.110	0.200	0.228
		p	0.996	0.063	0.114		0.533	0.408	0.163	0.083
Phosphate (mg/dL)		rho	0.076	0.279 (0.019-0.503)	-0.010	0.083	1.000	-0.504 (-0.676 to -0.279)	0.153	-0.021
		p	0.564	0.031*	0.940	0.533		<0.001**	0.284	0.871
25(OH)D (ng/mL)		rho	0.134	-0.099	0.161	0.110	-0.504 (-0.676 to -0.279)	1.000	0.062	0.086
		p	0.308	0.453	0.220	0.408	<0.001**		0.663	0.512
Adenoma max diameter (mm)^1^		rho	0.120	0.728 (0.561-0.839)	0.053	0.200	0.153	0.062	1.000	0.678(0.488-0.806)
		p	0.400	<0.001**	0.712	0.163	0.284	0.663		<0.001
PFi		rho	0.119	0.921 (0.869-0.953)	0.416(0.173-0.61)	0.228	-0.021	0.086	0.678(0.488-0.806)	1.000
		p	0.365	<0.001	<0.001	0.083	0.871	0.512	<0.001**	

**Figure 4:**
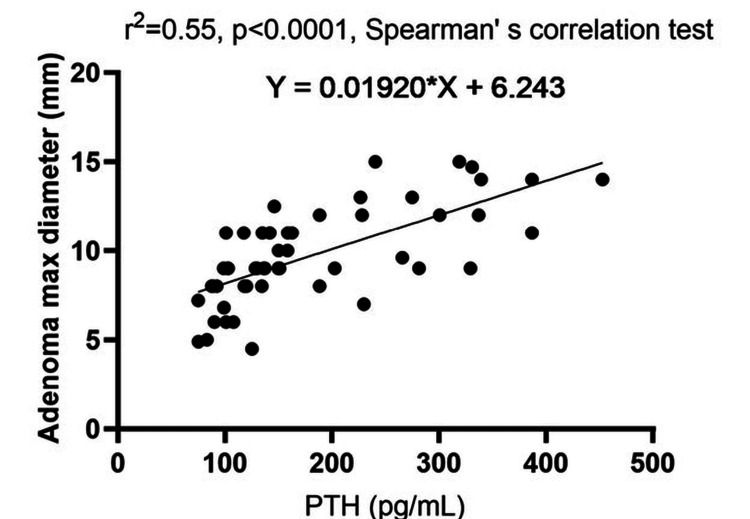
Correlation graphic between adenoma size and PTH level PTH: parathyroid hormone; r^2^: Spearman's correlation coefficient square

Figure 4 shows the significant linear correlation between adenoma maximal diameter and PTH level.

**Figure 5:**
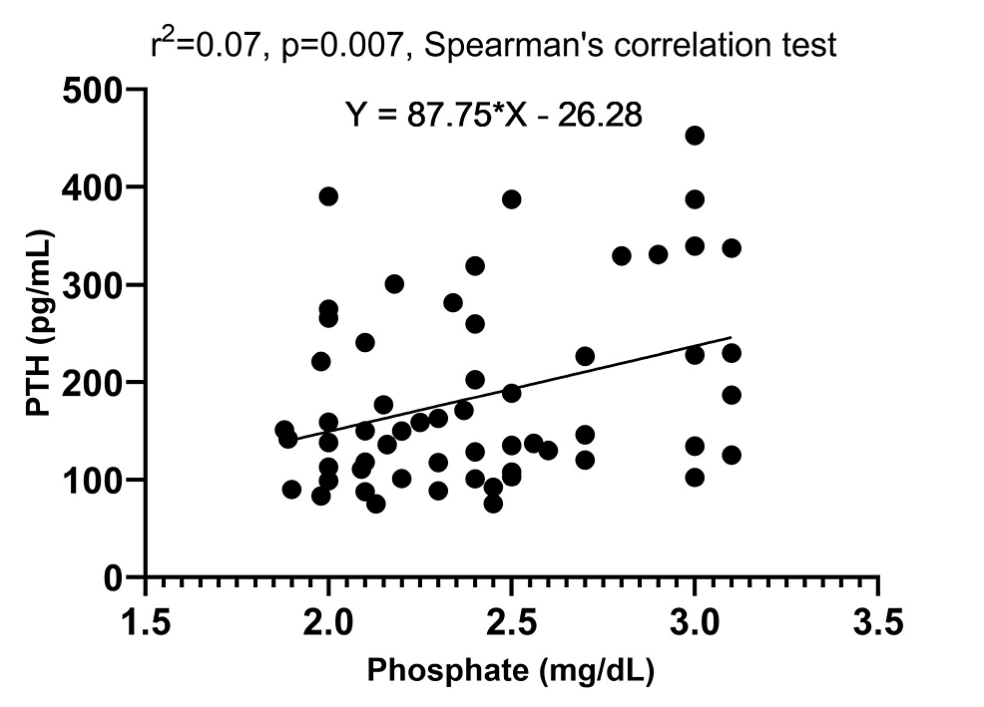
Correlation graphic between PTH and phosphate level PTH: parathyroid hormone; r^2^: Spearman's correlation coefficient square

Figure 5 shows the correlation between PTH and phosphate level.

**Figure 6:**
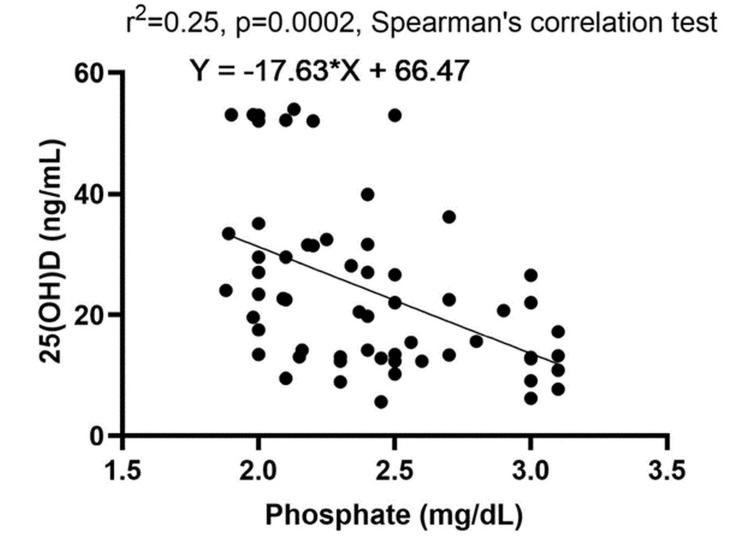
Correlation graphic between 25-hydroxyvitamin D and phosphate level r^2^: Spearman's correlation coefficient square

Figure 6 shows an inverse linear correlation between 25(OH)D and phosphate levels.

**Figure 7:**
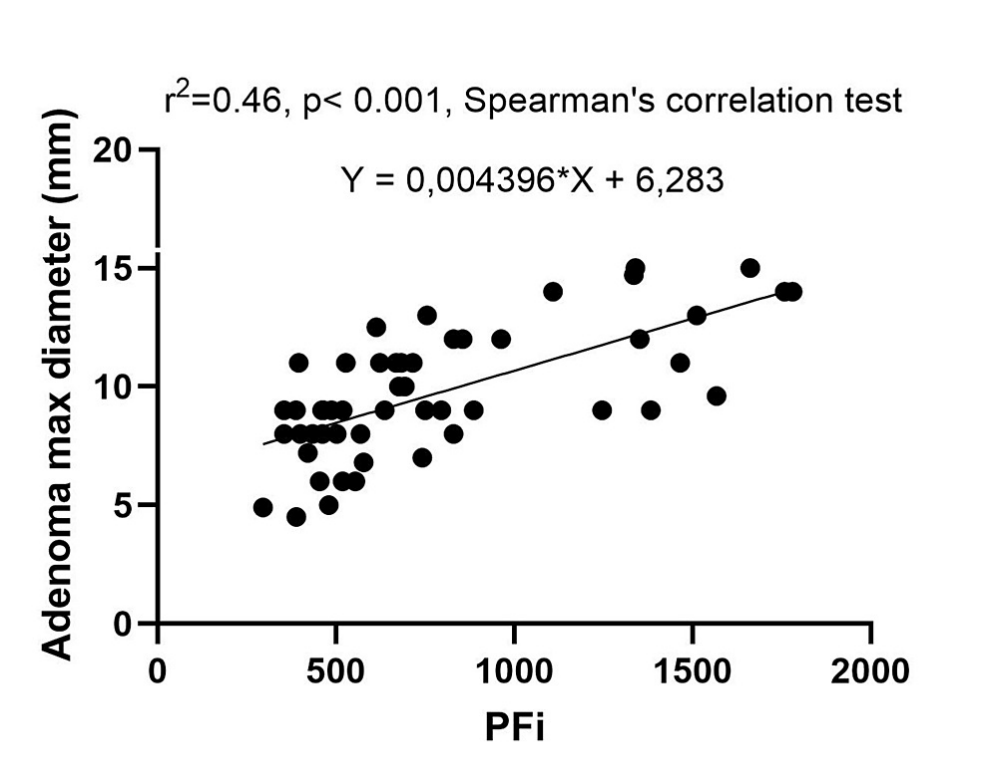
The PFi and adenoma size correlation graphic PFi: parathyroid function index; r^2^: Spearman's correlation coefficient square

 The PFi correlation with the adenoma size is presented in Figure 7.

Our binary logistic regression analysis investigates the predictors of fracture presence using age, adenoma size, PTH level, albumin-corrected calcemia, and 25(OH)D level as covariables. The results are presented in Table 5.

**Table 5:** Binary logistic model for fracture risk PTH: parathyroid hormone; B: estimated coefficient; SE: standard error; Wald: Wald statistic; df: degree of freedom; exp (B): exponentiated coefficient or odds ratio

	B	SE	Wald	df	p	Exp (B)
Age (years)	-0.022	0.045	0.246	1	0.620	0.978
PTH (pg/mL)	-0.013	0.010	1.902	1	0.168	0.987
Albumin-corrected calcium (mg/dL)	-0.607	0.602	1.019	1	0.313	0.545
25(OH)D (ng/mL)	-0.218	0.115	3.586	1	0.058	0.805
Adenoma max. diameter (mm)	0.481	0.372	1.670	1	0.196	1.618
Constant	6.699	6.313	11126	1	0.289	811.861

## Discussion

PHPT is typically characterized by elevated PTH and high calcium levels. However, there are variations of PHPT that present differently, such as NPHPT and cases associated with low vitamin D levels, representing a significant diagnostic difficulty in differentiating from secondary hyperparathyroidism. In our study, 51.66% (31/60) of the patients were normocalcemic, and 46.66% (28/60) presented deficient 25(OH)D vitamin level. Compared to other studies [12,13], NPHPT was more frequent, while PHPT associated with vitamin D deficiency [2] presented a similar prevalence. The explanation could be the exclusion by the majority of researchers from their PHPT patient cohort those with normocalcemia caused by low vitamin D level, considering this form secondary HPT.

The concept of NPHPT was first described in detail in 2003 [14], and although it has evolved from the notion of resistance to PTH to the subclinical form of HPTH [15], its connection with vitamin D deficiency is not clarified even after 20 years. The potential underlying mechanisms are multiple including  the stimulation of the enzyme 1-alpha-hydroxylase (encoded by *CYP27B1*) in the kidneys which leads to a higher rate of hydroxylation of 25(OH)D, resulting in lower circulating levels of 25(OH)D [16]. However, the difference between serum 25(OH)D and 1,25(OH)2D levels is a thousandfold, so this might not be the sole explanation. Due to the increased 1,25(OH)2D levels, the expression of 24-hydroxylase enzymes is also induced, which might accelerate the catabolism of the circulating form of 25(OH)D vitamin. Further, some studies suggest that PHPT may be associated with lower levels of vitamin D binding protein (DBP), which carries vitamin D metabolites in the blood. Lower DBP levels can reduce the total and free concentrations of 25(OH)D [17]. In rare cases, mutations or polymorphisms of calcium-sensing receptors coexist with PHPT being able to influence calcemia. When we compared 25(OH)D levels in NPHPT and HPHPT patients, a significantly lower level of 25(OH)D was found in normocalcemic patients. A recent study [11] has highlighted several advantages of the new biomarker of PHPT. PFi has been shown to have higher sensitivity and specificity compared to other diagnostic indices like the Wisconsin index and the calcium/phosphate ratio. The PFi demonstrated a sensitivity of 96.9% and a specificity of 97.6% for PHPT at a cutoff value of 34, making it a robust tool for distinguishing between PHPT and secondary hyperparathyroidism. In our study, we found that PFi was elevated in both the NPHPT and PHPT groups, indicating true PHPT. However, when eucalcemia was present, PFi was significantly (p=0.003) lower (568, IQR=279) compared with hypercalcemic patients (855, IQR=888). This is an important finding, as it suggests that in the presence of vitamin D deficiency, even if a person has a condition that should cause hypercalcemia, blood calcium levels might remain normal. This is because vitamin D deficiency impairs calcium absorption from the intestines. This "masking" of hypercalcemia by vitamin D deficiency is a key factor that could potentially lead to a delay in diagnosing PHPT if parathyroid function is not correctly evaluated. Moreover, vitamin D deficiency-induced compensatory secondary hyperparathyroidism might be a contributor to adenoma growth and parathyroid independence [18]. It's clear that further investigation and understanding of this phenomenon are needed because vitamin D supplementation might also have an impact on the clinical manifestation of PHPT. As both vitamin D and PTH are calciotropic hormones that increase serum calcium concentration, it is necessary to determine whether vitamin D supplementation is safe for individuals with known PHPT [19]. On the other hand, the benefits of vitamin D repletion in PTPH are multiple, as this preoperative intervention may limit disease progression and reduce gland size and postoperative PTH elevation [19]. Nevertheless, although there is some evidence that vitamin D repletion does not aggravate hypercalcemia or hypercalciuria, PHPT patients should be closely monitored because large studies are still lacking [20-22]. 

The comparison between fracture risks in NPHPT and hypercalcemic PHPT is an intricate and crucial topic. Studies show that both conditions pose a significant fracture risk, albeit with differing impacts on bone health [9,23]. In NPHPT, elevated PTH is the primary driver of bone turnover, while in hypercalcemic PHPT, both PTH and elevated calcium levels contribute to bone resorption. The absence of hypercalcemia in NPHPT may result in slightly less aggressive bone loss compared to hypercalcemic PHPT. Nevertheless, the overall fracture risk remains elevated due to the persistent effects of high PTH levels. Our study revealed that NPHPT did not show a higher incidence of osteoporotic DXA values than HPHPT, yet there was a significantly higher number of fractures present in NPHPT. An explanation could be the underdiagnosis of the NPHPT forms, and consequently, patients may suffer from prolonged exposure to elevated PTH levels, leading to significant bone loss and a higher fracture risk over time.  In NPHPT with concurrent vitamin D deficiency, the lack of sufficient vitamin D masks the hypercalcemia that would typically be present due to elevated PTH. This masking can delay diagnosis and treatment, allowing for prolonged periods of high bone turnover and increased fracture risk. In our study in 25(OH)D-deficient patients, NPHPT frequency and fracture number were significantly increased.  

Patients with hypercalcemic PHPT have a well-documented higher incidence of nephrolithiasis. Studies indicate that about 15-20% of patients with hypercalcemic PHPT develop kidney stones [9]. Other studies evidenced that NPHPT patients with higher urinary calcium excretion had a risk of nephrolithiasis like those with hypercalcemic PHPT [24,25]. The incidence of nephrolithiasis in NPHPT is less well-studied compared to hypercalcemic PHPT, but it is recognized that these patients also have an increased risk of kidney stones. Studies suggest that the risk, while present, might be lower compared to hypercalcemic PHPT due to the lack of persistently elevated serum calcium [26]. In our study, no differences were found between the prevalence of nephrolithiasis in the normocalcemic and hypercalcemic groups, further emphasizing the importance of our findings. 

PHPT, a condition often associated with parathyroid adenoma, is influenced by a range of factors, including genetic predisposition and biochemical factors (such as vitamin D status and PTH but not calcium levels) [27]. Our comprehensive study, which meticulously examined vitamin D status and calcium levels, found no significant differences in adenoma size among the various groups.

The adenoma size strongly correlated with PTH levels (rho=0.728, p<0.001) and PFi (rho=0.678, p<0.001), but PTH levels did not correlate with the corrected calcium levels (rho=0.206, p=0.114). An Australian study published in 2020, which enrolled patients with PHPT (n=555), found a strong positive correlation between preoperative PTH levels and parathyroid adenoma weight (rho=0.602, p<0.001). They proposed a formula for predicting adenoma weight based on preoperative calcium-PTH levels and age. However, the data in the literature are contradictory. Some studies have not found preoperative biochemical findings sufficient for predicting the size of parathyroid adenomas [28]. Based on our findings, we suggest using both PTH levels and PFi as an indicative marker for adenoma size prediction.

We found a significant correlation between PTH and phosphate levels (rho=0.279, p=0.031). The elevation in phosphate levels appears to trigger PTH synthesis (a key mechanism in chronic kidney disease-associated hyperparathyroidism). Anin vivo experiment demonstrated a significant PTH increase following phosphate infusion in dogs [29]. This PTH-phosphate correlation can be interpreted by transitivity: PTH negatively correlates with vitamin D levels, and vitamin D negatively correlates with phosphate levels, leading to a positive correlation between PTH and phosphate levels. Vitamin D deficiency may exacerbate the PTH-phosphate correlation, as it disrupts the negative correlation between PTH and vitamin D, leading to a stronger positive correlation between PTH and phosphate levels. This finding highlights the possibility that PHPT in the vitamin D-deficient group also has a secondary component. In our study, the correlation between 25(OH)D and phosphate levels was negative and moderate (rho=-0.504, p<0.001). Phosphate absorption in the small intestine is mediated by vitamin D through the sodium-dependent phosphate transporter type 2B. Low phosphate levels can stimulate 1-alpha hydroxylase activity independent of PTH. A study including children found similar results, showing a significant negative association between 25(OH)D vitamin and phosphate levels [30].

In our binary logistic regression analysis, we aimed to identify predictors of fracture presence and found an important result. The coefficient for vitamin D level was -0.218 with a p-value of 0.058, suggesting a potential association between lower vitamin D levels and higher odds of fractures in PHPT patients. Although this result may not have reached the conventional threshold for statistical significance, it does indicate a trend that could have significant implications for future research in bone health and fractures in PHPT.

The retrospective and observational nature of this study, the fact that patients were enrolled in one facility, and the absence of any intervention are among the limitations of this study. Moreover, the gender ratio is skewed towards females. Unfortunately, body mass index, another key parameter that would add value to vitamin D deficiency interpretation, is missing. It is also possible that the sample size is relatively small determining a wide CI, which limited the study's power and generalizability. However, the wide CI does not necessarily invalidate our findings, but it highlights the need for further research with a larger sample size to obtain more precise estimates and better understand the true effect size.

## Conclusions

In our study, PHPT was equally present in normocalcemic and hypercalcemic forms. We found that vitamin D deficiency plays a significant role in the clinical feature of PHPT. It influences the albumin-corrected calcium levels and bone health leading to an increased risk of fractures. Notably, PTH level and PTH-derived PFi correlate with adenoma size, making parathyroid hormone a more reliable predictor of the disease than calcium levels or vitamin D levels. Further research is needed to understand the interactions between PTH, vitamin D, and phosphate levels in PHPT to improve diagnosis and treatment strategies. In summary, this study emphasizes the need for careful evaluation of vitamin D status, PTH, calcemia, and phosphatemia in PHPT patients, as these factors significantly affect the clinical presentation, risk of fractures, and potential for delayed diagnosis.
